# Academy of Stomatology

**Published:** 1901-04

**Authors:** 

**Affiliations:** Academy of Stomatology


### ACADEMY OF STOMATOLOGY.

A REGULAR meeting of the Academy of Stomatology, of Philadelphia, was held at the rooms of the Academy, 1731 Chestnut Street, on the evening of Tuesday, January 22, 1901, the President, Dr. J. T. Lippencott, in the chair.

A paper was read by Dr. Emma E. Musson, upon the subject "Development of the Maxillary Sinus."

(For Dr. Musson's paper, see page 213.)

### DISCUSSION.

*Dr. Cryer.*—I have been much interested in Dr. Musson's paper, and I endorse what she has said, with a few exceptions. As to the diagnosis of enlarged antrum by observation of the contour of the face, I wish to state that when I heard Dr. Musson speak of this subject once previously, I looked for a skull presenting this characteristic, and having found one, I made a cross-section, expecting to find an enlarged antrum on the side presenting the greatest enlargement with a small antrum upon the depressed side; but I found the reverse to be true, and that the larger antrum was on the depressed side, and the smaller antrum on the other. Upon examining the thickness of the bones, it was observed that the prominent anterior wall was composed of thick bone, while upon the depressed side the bony tissue was thin. This, of course, is an exception, and, as a rule, you will find the antrum as shown in the illustration.

In the comparative anatomy of the dog and the cat the anterior and middle ethmoidal cells, including the maxillary sinus, seemed to have one common opening, although not so marked as in this illustration before us. There are well-marked cases in human skulls, where a slit-like opening extends from the maxillary sinus through the ethmoidal cells into the frontal sinus.

One of Dr. Musson's illustrations shows the division of the maxillary sinus vertically. In cutting perhaps five hundred or six hundred skulls, I have failed to find an antrum that was completely divided by either bony or membranous septa. A bony or membranous septum may be found, but there is always a communication through this septum. Dr. Musson spoke of the sinus having two openings, one from each division. I have found cases where the posterior one opened into the superior meatus, and in those cases took it for granted that the posterior division belonged to the orbital process of the palate bone, there being a cell in that process. Several skulls were found where this cell had been enlarged, extending downward and backward and curving around the maxillary sinus, and I thought this was a true division of the maxillary sinus, but upon looking for the outlet, I found it to open into the superior meatus. Therefore this is not a division of the maxillary sinus, but an enlarged cell of the orbital process of the palate bone. I am still hoping to find a case of true division of the maxillary sinus.

The doctor passed around a beautiful specimen of the infraorbital sinus. I entered into a description of this at the American Dental Association in 1895, and spoke of a case in which the sinus was enlarged, and where the nerve, instead of passing out through the solid bone, passed downward, forward and across the anterior superior corner of the antrum.

It has been a great pleasure to have Dr. Musson with us this evening, and her paper has been most instructive and interesting.

*Dr. Schamberg.*—I wish to state my appreciation of the paper, inasmuch as the doctor has given us means of recognizing pathological conditions of the antrum during life. We can easily find these conditions on post-mortem sections, but it is highly important that some one endeavor to make clear to us how we are to recognize these various conditions during life, and I must say the paper this evening goes a little farther in this direction than any that I have ever heard. There are few things which are of more decided advantage to us as dentists than an understanding of the proper point at which to open the antrum in diseases of that cavity. I must take exception to the opening through the roof of the mouth, inasmuch as the tongue would tend to force food up through the opening, and therefore I would prefer to enter the antrum through the anterior wall. It is highly essential to remember that a small opening into the antrum is of little advantage. You so frequently find conditions in the antrum that cannot be cured by syringing alone. The antrum often requires curettage. I had a case a few months ago where I expected to find a discharge of pus, but upon opening the antrum this did not prove to be the case. A few days afterwards, however, I got a considerable discharge of pus, and there was evidently an abscess on the superior part of the anterior wall, and this illustrates the fact that it is highly essential to have a large opening, and a large opening in the roof of the mouth would interfere considerably with deglutition.

*Dr. Curry.*—Dr. Musson spoke of resorption of the bone, and I wish to state that I once had a patient, about seventy years of age, who had no teeth posterior to the cuspids, and who complained of a great deal of pain in the antral region. I passed a bistoury through the gum, and it dropped directly into the antrum without any resistance; there did not appear to be any bone whatever. I will ask Dr. Musson if she has ever noticed that degree of resorption in any of her cases.

*Dr. Musson.*—With regard to Dr. Cryer's remark that the bulging of the bone could not be considered indicative of enlargement of the antrum, we would, of course, be aided in our diagnosis by the transillumination test.

As to the cell in the orbital process of the palate bone, I would consider that as a prolongation of the antrum. I know these cells do sometimes exist, and if I should find an apparent division of the antrum opening into the superior meatus, I should call that an ethmoidal cell, and not a true division of the antrum. After all, these sinuses are a continuation of cells, and are named according to the region in which they are found. It is almost impossible in some of my specimens to tell the difference between the ethmoidal cells and the sphenoidal cavity.

As to Dr. Schamberg's remark in regard to opening the antrum, I did not recommend opening through the roof of the month, I merely said it was possible. A very large opening into the antrum is needed in order to treat it properly, and I almost always operate in the canine fossa, unless, indeed, the first or second molar is gone, when I open up through that alveolar process. If I suspect a condition of caries, I have the tooth removed and open up through its alveolus. With regard to the case Dr. Schamberg mentions, in which he obtained no discharge of pus on opening the antrum, the discharge occurring a few days later, I have explained these cases by the fact that the secretion is inspissated, dry, and adherent, but after two or three days you get a marked purulent secretion due to the dislodgement of old dried secretions. In operating where we have growths, I believe the Caldwell-Lue operation is the best. An opening is made through the canine fossa and one through the inferior meatus. That in the canine fossa is later closed and the opening into the inferior meatus left open for drainage. This gives the best results where we desire curettage and where there are polypoid growths.

As to absorption of the antral floor occurring to such an extent as to leave no process between the root of the teeth and the antrum itself, in a case of this kind I should be inclined to think that when the teeth were removed, the floor of the antrum came away with them.

*Dr. Kirk.*—As this seems to be the evening for consideration of surgical matters, I would like to get opinions relative to operation in cases of abnormal protrusion of the lower jaw, where a resection is made, taking from the jaw on each side a V-shaped section and resetting the jaw in a normal position, as described first by Hullihen, of Virginia, and recently by Whipple, of St. Louis. Quite recently there have been sent to me models of a case where protrusion or abnormal growth forward of the lower jaw has taken place, making a very ugly deformity which the patient desires to have corrected, a case which I should say was intractable so far as any ordinary method of mechanical treatment is concerned, especially at the age of the patient. The point I desire to raise is whether, in an operation of this kind, involving a complete section through the body of the bone and removal of a large portion of it, involving a section of the nutrient vessels and nervous connections of the teeth, it would not necessarily destroy the nervous and nutrient supply of the teeth anterior to the section? Or, to put it in another way, what are the chances for a restoration of the nervous and vascular relationships of the teeth anterior to the field of operation? I ask for information, so that I may give advice to the man who asks the question.

*Dr. Cryer.*—About four months ago Dr. Roberts asked my opinion of an operation of this kind, and I told him I would like to see such an operation, but did not care to undertake one. A young man who has been going the rounds of surgeons wishing such an operation to be done finally reached me. I told the boy I would not undertake the operation under any consideration. My answer to Dr. Kirk is that I would not operate for a condition of this kind. It is only undertaken to improve the appearance, and there is little in the appearance of a man. If he accomplishes something in life, his appearance will not be thought of.

The teeth anterior to the section would, I believe, lose their sensation through severance from the source of nervous energy, but with regard to the nourishment I am not so certain. If the operation were performed carefully there would be a collateral circulation sufficient to preserve the life of the teeth. The teeth would not respond to heat or cold, but it is not necessary that the pulps should die. Surgeons are constantly making resections of the inferior dental nerve between the foramen ovale and the inferior dental canal, and the nerve has been removed along the greater portion of the canal, yet the teeth have not lost their vitality, and therefore I believe that in an operation such as Dr. Kirk questions, the vitality of the teeth would not be destroyed. Perhaps the nerve might regenerate, and even sensation be restored. My chief objection to the operation is the great risk of infection and necrosis of the bone.

*Dr. Kirk.*—I think Dr. Cryer has made out a pretty good case in favor of this operation, after all. He objects to it on general principles, and yet, as a matter of fact, he says he thinks the circulation can be re-established by collateral circulation, and that even a reestablishment of nervous supply is possible. Why, then, should he take the ground that the operation should not be done? I know nothing about it practically, but ask for information.

*Dr. Cryer.*—We sometimes find, in the simple extraction of a tooth, that there will be a separation of the jaw, leading to great deformity and bad consequences. Now, when you come to take a V-shaped piece out of each side of the jaw, it is a very nice operation to cut these pieces out alike on both sides. I do not care what instrument you use. Experiment upon the cadaver, and see how difficult it is to get perfect apposition of the cut surfaces. Some time since we had a case of pathological separation of the lower jaw, where the inferior dental nerve and vessels and a great portion of the bone, extending from the second molar to the canine tooth, were destroyed; but by the ingenuity of Dr. Gaylord a bridge was made, extending from the second and third molars forward, capping all the anterior teeth and uniting the parts; the necrosed bone was cut away with a surgical engine. That splint or bridge remained on from June until the following January. The parts are now united with true bone, no depression being evident on the outer, inner, or lateral surfaces. On the upper surface, however, the alveolar processes are lacking, and will never be reproduced. The sensation is already returning to the lip and other tissue anterior to the injured part. Remember that this case was on one side only, and that there was a chance to hold the parts in their true position, and that the periosteum was only partly removed. As for cutting through both sides, I would not want to undertake it. Perhaps I am getting too old for an operation of this kind, and some of the younger men who have not seen quite so much surgery in that locality would undertake it.

*Dr. Schamberg.*—I wish to suggest an operation which I think would take the place of the one Dr. Kirk has mentioned, and that is, instead of taking out a V-shaped piece, to make a median section through the symphysis, so bevelling the edges that when brought together there will be a retraction of the anterior portion of the jaw, sacrificing, if need be, the central incisors. This would likely require spreading of the posterior portion of the jaw in order to get retraction of the anterior part. We would necessarily have to change the occlusion in the first operation, and I imagine the occlusion in a jaw of that kind is anything but perfect.

*Dr. Kirk.*—With regard to the point Dr. Cryer has raised,—namely, the great difficulty in making the section so as to get perfect apposition of the cut ends of the bone, I would call attention to the fact that in the cases that have been operated upon it was done by using parallel saws and cutting so that the surfaces were exactly adaptable. A very rigid means of fixation was then applied, and firm union was secured in ten days.

*Dr. Cryer.*—Mr. President, I would like to move that a vote of thanks be extended to Dr. Musson for her paper this evening.

Carried.

Adjourned.

OTTO E. INGLIS, D.D.S.,
*Editor Academy of Stomatology.*

